# Looking for the LOAEL or NOAEL Concentration of Nickel-Oxide Nanoparticles in a Long-Term Inhalation Exposure of Rats

**DOI:** 10.3390/ijms22010416

**Published:** 2021-01-03

**Authors:** Boris A. Katsnelson, Ivan N. Chernyshov, Svetlana N. Solovyeva, Ilzira A. Minigalieva, Vladimir B. Gurvich, Irene E. Valamina, Oleg H. Makeyev, Renata R. Sahautdinova, Larisa I. Privalova, Anastasia E. Tsaregorodtseva, Artem V. Korotkov, Eugene A. Shuman, Vladimir G. Panov, Marina P. Sutunkova

**Affiliations:** 1 The Yekaterinburg Medical Research Center for Prophylaxis and Health Protection in Industrial Workers, 30 Popov Str., 620014 Yekaterinburg, Russia; chernyshov@ymrc.ru (I.N.C.); solovyevasn@ymrc.ru (S.N.S.); Ilzira-Minigalieva@yandex.ru (I.A.M.); gurvich@ymrc.ru (V.B.G.); sahautdinova@ymrc.ru (R.R.S.); privalovali@yahoo.com (L.I.P.); panov.wlad1mir@yandex.ru (V.G.P.); marinasutunkova@yandex.ru (M.P.S.); 2 Central Research Laboratory, The Ural State Medical University, 17 Klyuchevskaya Str., 620109 Yekaterinburg, Russia; ivalamina@mail.ru (I.E.V.); larim@mail.ru (O.H.M.); Tsaregorodtseva@mail.ru (A.E.T.); cardiovektor.inbox@gmail.com (A.V.K.); evgenyshuman@gmail.com (E.A.S.); 3 Institute of Industrial Ecology, The Urals Branch of the Russian Academy of Sciences, 620990 Ekaterinburg, Russia

**Keywords:** nickel oxide nanoparticles, inhalation exposure, pulmonary and systemic toxicity, genotoxicity, allergic sensitization

## Abstract

Rats were exposed to nickel oxide nano-aerosol at a concentration of 2.4 ± 0.4 µg/m^3^ in a “nose only” inhalation setup for 4 h at a time, 5 times a week, during an overall period of 2 weeks to 6 months. Based on the majority of the effects assessed, this kind of exposure may be considered as close to LOAEL (lowest observed adverse effect level), or even to NOAEL (no observed adverse effect level). At the same time, the experiment revealed genotoxic and allergic effects as early as in the first weeks of exposure, suggesting that these effects may have no threshold at all.

## 1. Introduction

Like many other metal oxides, nickel-oxide nanoparticles (NiO-NPs) are of special toxicological interest considering that this species is not only being produced for various industrial applications but also makes up a substantial proportion in the particle size distribution of condensation aerosols generated by metallurgical and arc-welding technologies. That is why NiO-NP toxicology has been the subject-matter of numerous experimental studies (Reference [1,2,3,4,5,6,7,8,9,10,11,12,13,14,15,16,17] and many others). However, the prevailing majority of these studies were performed in vitro on cultured cells or, in some cases, in vivo on daphnia and drosophila, while the experiments on laboratory rodents used mostly single-shot or repeated intratracheal instillations and, occasionally, oral administration. In the few inhalation experiments we have found in the literature, exposure lasted no longer than 4 weeks [18,19,20,21].

Thus far, the first, and, to the best of our knowledge, the only, study on outcomes produced by long-term inhalation exposure to NiO-NP is ours [22]. In this inhalation study, rats were exposed to nickel-oxide nanoparticles (NiO-NP) at 0.23 ± 0.01 mg/m^3^ (230 ± 10 µg/m^3^) for 4 h a day 5 times a week for up to 10 months. The rat organism was found to respond to this impact with changes in the cytological and some biochemical characteristics of the bronchoalveolar lavage fluid (BALF) typically associated with the deposition of cytotoxic particles in the lower airways. This effect came along with a paradoxically little-pronounced pulmonary pathology due to a rather low chronic retention of nanoparticles in the lungs. Various manifestations of systemic toxicity were also observed, including damage to the liver and kidneys; a likely allergic syndrome as indicated by some cytological signs; transient stimulation of erythropoiesis; and translocation of NiO-NPs to the brain from the nasal mucous membrane along the olfactory pathway. Against this picture of mild to moderate chronic toxicity of nickel, its in vivo genotoxic effect assessed by the degree of DNA fragmentation in nucleated blood cells (the RAPD test) was distinct, tending to increase with the length of the exposure period.

The level of experimental exposure adopted in that study may appear very high in relation to experimental modeling of 8-h working day human exposure if we recall that OEHHA established REL for such conditions at as low as 0.06 µg/m^3^ (60 ng/m^3^) in Ni content terms. (This value was validated in experiments on mice, the critical organ being the respiratory system only). Meanwhile, the above concentration of nanoparticles in [22], also expressed in Ni-content terms, was equal to about 180 µg/m^3^.

However, the allowable nickel oxide concentrations in the air for 8-h exposure adopted by other US agencies for similar contexts are much higher: ACGIH TLV 0.2 mg (i.e., 200 µg) Ni/m^3^ (inhalable fraction); OSHA PEL 1 mg (i.e., 1000 µg) Ni/m^3^. However, none of the three regulations specifies whether they are also applicable to nickel-containing nanoparticles, NiO-NP in particular. Meantime, it is widely assumed and, in many cases, experimentally proved that nanoparticles are significantly more noxious than larger particles of the same chemical nature (for instance, Reference [1], and many others). Therefore, even when a presumably safe exposure level for an aerosol comprising micrometric particles has been established, a question remains whether the same level would be sufficiently safe for respective nanoparticles too.

It therefore remains important to continue conducting chronic inhalation experiments (assessing not only the respiratory but also multiple-vector systemic toxicity of NiO-NP) in order to identify the air concentration of this substance that could serve a starting point for establishing hygienic parameters, such as the above-mentioned REL, PEL, and TLV, or the MAC (Maximum Allowable Concentration) in Russia and some other countries of the former Soviet Union for this substance in workroom air. In Russia, this kind of starting point is the so-called “threshold” concentration under chronic exposure, the meaning of which is close to the internationally widespread term LOAEL (lowest observed adverse effect level). Both terms imply the lowest concentration of a substance found by experiment that causes an adverse alteration of morphology, function or capacity. Although these parameters are not rigorous statistically, their at least rough estimation is a very important stage in the establishment of safety standards. Along with this, use is also made of the experimentally established NOAEL (no observed adverse effect level), in which statistical uncertainty is undoubted, as well.

Given the findings of the above study [22], we have conducted a new experiment of similar design but with 2 orders lower NiO-NP concentration, namely 0.0024 ± 0.0004 mg/m^3^ (2.4 ± 0.4 µg/m^3^) of particles by mass. It should be noticed that our approach is based on comparing effects of different exposure levels observed in different experiments carried out not in parallel. To conduct simultaneous long term experiments in several nose-only inhalation device with computerized control of NP-concentration would be a too laborious and very expensive task, and it is no wonder that such designs are very difficult to find in nanotoxicological literature.

## 2. Results and Discussion

### 2.1. Pulmonotoxicity

As follows from the data presented in Table 1, the shifts in the cytological characteristics of the BALF 24 h after the final inhalation exposure to nanoparticles were of usual kind: the total cell count increased due to the recruitment of both alveolar macrophages (AM) and neutrophil leukocytes (NL) with an increase in the NL/AM ratio in comparison with the control group (although not all differences were statistically significant). It is noteworthy, however, that these shifts were, in general, less pronounced than in the similar experiment conducted with a considerably higher concentration of the same nanoparticles [22].

Assuming that these shifts were determined mainly by the defensive response to the last portions of particles deposited in the lower airways, it is no wonder that we neither expected nor found them to increase with increasing the overall duration of the exposure period. Indeed, the cytological characteristic of the BALF after the 2-week and 6-month exposure periods was in principle the same. Meanwhile, if the neutrophilic response of the lower airways reflected not so much the dose-dependent functioning of the physiological mechanisms of lung clearance [23,24] as the development of an inflammatory process in them, one would expect its gradual increasing. The absence of any chronic inflammation is also evidenced by the absence of usual unambiguous and statistically significant shifts in the biochemical characteristics of the BALF (Table 2), while such shifts were observed in the previous experiment [22]. In the meantime, the 6-month exposure period corresponds to 20% of lab rat’s normal 2.5-year lifespan, i.e., it is equivalent to 14 years in terms of 70-years human life expectancy. Thus, this exposure could be justly considered as a long-term one.

**Figure 1:**
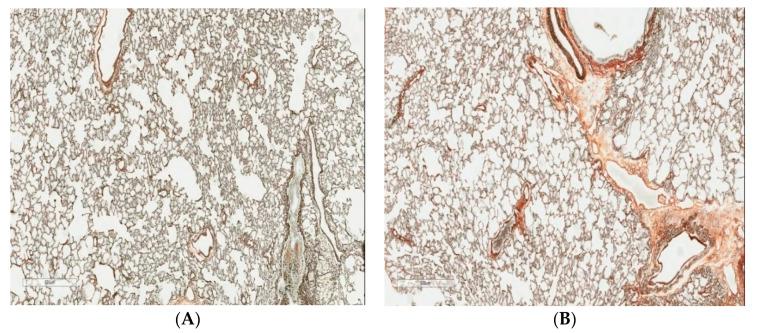
Rat lungs (**A**) after 6-months inhalation exposure to NiO-NPs and (**B**) in the control group of the same exposure period. Gomori’s silver impregnation, magnification × 600.

In toto, judging from all the BALF indices considered above, the NiO-NP concentration tested in the current experiment is, for impact on the airways, even lower than LOAEL. The same is evidenced by the absence of noticeable differences between the exposed and sham-exposed rats till the very end of the 6-month exposure period judging by the morphological picture of the lungs examined histologically (Figure 1).

### 2.2. Systemic Toxicity

All the organ and system indices assessed in this experiment are presented in the Supplements (Tables S1 and S2). It is noteworthy that out of the several dozens of functional indices only four (bar the DNA fragmentation coefficient of nucleated blood cells and some signs of allergic sensitization) displayed a statistically significant difference from the control (Table 3). Specifically, a considerable reduction in brain and kidney mass and endogenous creatinine clearance were noted only towards the end of the 6-month exposure period. The increase in the proportion of reticulocytes discovered as early as after the first two weeks and increasing with exposure length was more persistent. All other hematological indices remained unchanged till the end of the experiment (The rather contradictory literature data on the impact of nickel poisoning on red blood were presented in our previous publication [22].). It should be noted, however, that the response of the blood to a hundred-times higher level of exposure to NiO-NP [22] was similarly paradoxical, while the total number of statistically significant shifts towards the end of the exposure period was about the same (six, also not counting the above-mentioned exclusions).

Table 3 also reveals a relatively low but statistically significant increase in the genomic DNA fragmentation coefficient in nucleated blood cells, which was observed already after the first two weeks of exposure when the dose of nickel retained in the organism could be but only negligible. It is worth noting that, whereas, in the control group of rats, this index was practically stable over the entire 6-months period, in the exposed group, it grew modestly but steadily from one exposure period to another. This may be thought to be associated with gradual growth of the cumulative internal dose of nickel (Unfortunately, for technical reasons were unable to determine the Ni contents of rat tissues and blood in this experiment, but in the previous one [22] it was elevated but little even for a higher exposure level.). Only towards the end of a period was the DNA fragmentation factor somewhat lower than at the corresponding time point in the previous experiment [22] involving a higher concentration of the same nanoparticles (0.5332 ± 0.0031) and similar control value (0.4247 ± 0.0006). It should be stressed that this important adverse effect of Ni was revealed also by other experimental investigations, both in vitro [25,26,27] and in vivo [28,29,30].

These results are consistent with the widespread assumption that toxicants carry a risk of genotoxic (and related carcinogenic) impact in a dose dependent but no-threshold character. From this standpoint, the concept of LOAEL/NOAEL is not applicable to these effects at all, and national or international safety standards proposed on its basis are merely corrected depending on the adapted policy in relation to the admissibility or inadmissibility of some or other levels of carcinogenic risk.

It is even more difficult to decide on the significance of some early signs of sensitization to nickel in exposed rats also discovered in our current experiment for the purpose of establishing hygienic safety standards (Table 3). Moreover, the allergic response of the organism to NiO-NP inhalation exposure of varying duration may be indirectly evidenced by the increase in the cellular percentage of eosinophils in the spleen tissue imprints. Note that under higher exposure levels this shift was more marked in spleen, and found also in the liver [22].

The possibility of a no-threshold character of allergic responses to toxic exposures is not discussed in the literature as broadly as the action of genotoxic carcinogens: however, this possibility is suggested by the well-known fact that sensitization may develop in humans under the action of very low doses of allergic agents (nickel, in particular). This fact also renders the LOAEL/NOAEL concept less definitive but does not preclude its use for establishing safety standards for other (non-allergic) effects. Thus, many of the officially adopted MACs in Russia are marked by the index “a”, pointing to the possibility of allergic reactions in individuals even under industrial exposures not exceeding the admissible level.

It should be noted that according to the data from [13], the early onset of an eosinophilic reaction in rat lungs to intratracheal instillation of NiO-NP may be associated with the direct release of intracellular eotaxin triggered by NiO-NP rather than with sensitization to nickel. However, in the inhalation experiment being discussed, we did not see this reaction judging by the lung imprints (Supplement Table S2).

In our previous study [22] with chronic inhalation exposure to NiO-NP at a higher concentration, as well as in under subchronic intraperitoneal intoxications with different metallic nanoparticles (e.g., Reference [1,2]), we observed noticeable histopathological changes in the liver, kidneys, spleen, brain and corresponding statistically significant shifts in some morphometric indices. On the contrary, in the current study the histological characteristic of the above organs appears to be identical for the exposed and control rats even towards the end of the 6-month experiment. At the same time, a few morphometric indices indicated an adverse effect of the toxic exposure. Thus, after the 3-month inhalation exposure to NiO-NP, the liver displayed a statistically significantly (*p* < 0.05) increased percentage of binuclear hepatocytes (4.47 ± 0.22 against 2.00 ± 0.20 in the control) and Kupffer cells (16.89 ± 0.82 against 12.06 ± 0.71), but, after six months of exposure, only the latter were elevated (12.94 ± 0.38 against 9.89 ± 0.63). In this case, however, we did not come across the most important index of damage to the liver often observed in our other experiments (including [22]), i.e., increased percentage of acaryotic hepatocytes.

The percentage increase in the length of proximal convoluted tubules with brush border loss typically associated with a toxic renal damage, in this experiment, took place only after the 6-month period of exposure (12.69 ± 1.83 against 7.86 ± 0.96 in the control), but complete tubular epithelium desquamation was not increased in length (1.68 ± 0.67 in the experiment against 2.61 ± 0.66 in the control). The glomerular diameter was not changed in any of the experimental exposure periods.

The morphometry of the histological spleen preparations showed a statistically significant increase in the planimetric red to white pulp ratio only after the 6-month exposure period (1.55 ± 0.07 against 1.26 ± 0.07 in the control, *p* < 0.05). However, after the 3-month period, there was, on the contrary, a statistically insignificant decrease in this index.

Thus, the results of histological examination of internal organs are generally consistent with the conclusion that the NiO-NP concentration in this experiment was close to LOAEL for chronic inhalation exposure.

## 3. Materials and Methods

Both the previous [22] and the present experiment were carried out on 3–4 month old outbred white female rats from our own breeding colony with an initial body mass of 180–230 g. When not being actually exposed, all rats were housed in conventional conditions, breathed unfiltered air and were fed standard balanced food. The experiments were planned and implemented in accordance with the “International guiding principles for biomedical research involving animals” developed by the Council for International Organizations of Medical Sciences (2012) and were approved by the Ethics Committee of the Ekaterinburg Medical Research Center Medical for Prophylaxis and Health Protection in Industrial Workers.

Airborne NiO-NPs were obtained from 99.99% pure nickel rods by sparking in the Palas DNP-3000 generator and fed into a nose-only exposure tower (CH Technologies, Westwood, NJ, USA) for rats placed into individual restrainers. A device of the same design obtained from the same supplier was used for a sham exposure of control rats; this device was being fed with air from the same compressor.

**Figure 2:**
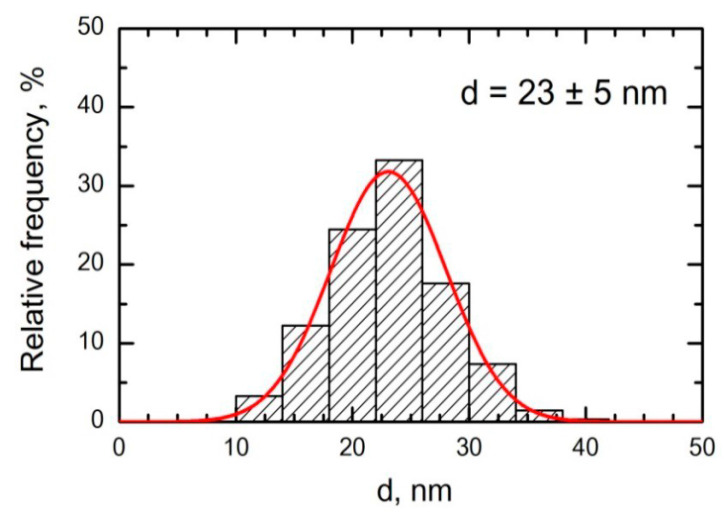
Particle size distribution function obtained by statistical processing SEM images of particles accumulated on a polycarbonate filter from the exposed rats’ breathing zone in the experiment with NiO-NP in the concentration 230 ± 10 µg/m^3^ [22].

Particles collected on a polycarbonate filter placed instead of one of the restrainers and inspected under a scanning electron microscope (SEM) had a spherical shape and the particle size distribution was restricted to the nanometric range. In the previous experiment this distribution (Figure 2) was estimated on a great number of filters and demonstrated the mean (±s.d.) diameter of nanoparticles equal to 23 ± 5 nm. In the present experiment, we examined but several samples and found that the size of nanoparticles was virtually the same.

The chemical identity of the NPs sampled on the filters was confirmed by Raman spectroscopy to be NiO.

Rats were exposed or sham-exposed in parallel for 4 h a day, 5 times a week for 2 and 4 weeks, 3 and 6 months. The mass of Ni retained on abovementioned filter was determined with an atomic absorption spectrometer ContrAA 700 (“Analytic Jena AG”, Jena, Thüringen, Germany) and translated into NiO mass and then into its air concentration as µg/m^3^. Mean concentrations for different exposure durations were (in µg/m^3^): 2.45 ± 0.97 for 2 weeks; 1.93 ± 0.45 for 4 weeks; 1.59 ± 0.30 for 3 months; 2.44 ± 0.36 for 6 months.

Twenty-four hours after the final exposure in each of the above-mentioned subperiods, we performed (a) broncho-alveolar lavage to obtain a fluid (BALF) for cytological and biochemical characterization and (b) the following toxicometric tests:

1. weighing of the body;
2. estimation of the central nervous system’s ability to induce temporal summation of sub-threshold impulses—a variant of withdrawal reflex and its facilitation by repeated electrical stimulations in an intact conscious rat;
3. recording of the number of head-dips into the holes of a hole-board (which is a simple but informative index of the exploratory activity frequently used for studying behavioral effects of toxicants and drugs), as well as of the number of squares crossed during the same time interval—as a measure of motion activity;
4. collection of daily urine for analysis of its output (diuresis), specific gravity (density), protein, total coproporphyrin, δ-aminolevulinic acid (δ-ALA), urea, uric acid, creatinine, and Ni content; and
5. sampling of capillary blood from a notch on the tail for examining the hemogram and hemoglobin content and for cytochemical determination of succinate dehydrogenase (SDH) activity in lymphocytes (by the reduction of nitrotetrazolium violet to formazan, the number of granules of which in a cell was counted under immersion microscopy).

The rats were then sacrificed by semi-decapitation, and blood was collected by exsanguination. The liver, spleen, kidneys, and brain were weighed. The biochemical indices determined from the blood included reduced glutathione (GSH), total serum protein, albumin, globulin, bilirubin, ceruloplasmin, malondialdehyde (MDA), alkaline phosphatase, alanine- and asparate-transaminases (ALT, AST), catalase, gamma glutamyl transferase, SH-groups, urea, uric acid, and creatinine.

All the routine clinical laboratory tests on blood and urine were performed using well-known techniques described in many manuals (for instance, [31]).

Liver, spleen, kidney, and brain tissue sections were prepared from 4 rats in each treated and control group of 3- and 6-month term for histological examination by hematoxilin-eosine staining and, where necessary, Periodic acid–Schiff (PAS), Nissl, or Perl’s stain. We used the Avtandilov’s planimetric ocular grid for morphometric characterization of the spleen. Liver and kidneys were characterized morphometrically using the programmed image-recognition system CellSens (Olympus, Ekaterinburg, Russia).

To estimate the allergic sensitization of the organism to nickel, 0.1 mL of blood was added to each of the two centrifugal test-tubes contain: 1 (experimental)—0.05 mL of normal saline with 2 µL of the allergen dissolved in it; 2 (control)—0.05 mL of normal saline without the allergic agent. Both test tubes were incubated for two hours at 37 °C. Then, 0.02 mL portions of each sample were transferred into two corresponding test tubes, each containing 0.4 mL of 3% acetic acid solution in water for RBC disruption. Absolute leukocyte counting was performed in a Goryaev counting chamber.

The result of the reaction was estimated by calculating the index by the formula:

(1) $$RSLL=\frac{\left(L_{control}-L_{exp}\right)}{L_{control}}\times 100\%$$

where L is the absolute number of leukocytes. The reaction is considered as positive for an index of 10% and higher. In the absence of sensitization, the working dose of the allergen should not cause lysis of intact leukocytes in numbers greater than in the control (allergen-free), and the average value of the index should not exceed 9.9%.

For determining specific Ig E, the blood serum of an exposed or control rat is preliminarily incubated at 37 °C for 60 min. The chemical allergen is diluted in normal saline to 4% (25-fold dilution). The serum to diluted allergen ratio for the purposes of ELISA is 1:1.

For the Random Amplification of Polymorphic DNA (RAPD) Test, blood samples were collected in special vessels cooled to −80 °C. To isolate DNA from the cells, we used a GenElute (Sigma, St. Louis, MO, USA) set of reagents in accordance with the manufacturer’s guidelines for use. The DNA content of the samples was determined spectrophotometrically (Ultraspec 1100 pro. Amersham Biosciences. Ltd., Amersham, UK) and they were then frozen and stored at −84 °C in a kelvinator (Sanyo Electric Co. Ltd., Moriguchi, Japan) until the beginning of the implementation of the RAPD test performed as in many previous experiments of ours (including Reference [22]). To characterize the degree of damage to DNA, we used the “coefficient of fragmentation” (Cfr), i.e., the ratio of total radioactivity of all tail fractions to that of the head.

Number of rats in each control and exposed group within a particular term of our investigation amounted to: 7–10 for BALF testing, 6–10 for organs weighting, blood analysis, biochemical indices of toxicity, cytomorphology of tissue imprints, 13–20 for physiological and immunological testing, RAPD and urine analysis—the total number of rats being 133.

## 4. Conclusions

For the majority of the respiratory and systemic toxic effects of long-term (lasting up to 6 months in total) inhalation exposure of rats to nickel-oxide nanoparticles that we assessed, the concentration of 2.4 ± 0.4 µg/m^3^ under five 4-h exposures 5 times a week may be regarded as close to LOAEL, or even to NOAEL.

At the same time, this inhalation impact was found to produce genotoxic and allergic effects as early as in the first weeks of exposure, which suggests that these effects may have no threshold.

## Figures and Tables

**Table 1:** Number of cells in the bronchoalveolar lavage fluid (BALF) obtained 24 h after the last 4-h inhalation exposure to nickel-oxide nanoparticles (NiO-NP) at 2.4 ± 0.4 µg/m^3^; (*x* ± *s.e.*).

Number of Cells (× 10^6^)				
Exposure Exposed to:	Total	Neutrophil Leukocytes (NL)	Alveolar Macrophages (AM)	NL/AM
2 weeks				
NiO-NP	3.03 ± 0.28 *	0.67 ± 0.14 *	2.36 ± 0.26 *	0.33 ± 0.08
Sham	1.75 ± 0.11	0.22 ± 0.07	1.54 ± 0.11	0.16 ± 0.06
4 weeks				
NiO-NP	3.37 ± 0.41 *	0.92 ± 0.28 *	2.44 ± 0.28 *	0.41 ± 0.13
Sham	1.67 ± 0.28	0.28 ± 0.08	1.38 ± 0.25	0.28 ± 0.13
3 months				
NiO-NP	5.04 ± 0.63	0.66 ± 0.17 *	4.36 ± 0.52	0.15 ± 0.03 *
Sham	4.38 ± 0.56	0.19 ± 0.04	4.18 ± 0.53	0.05 ± 0.01
6 months				
NiO-NP	4.86 ± 0.98	0.85 ± 0.19 *	4.01 ± 0.85	0.23 ± 0.06 *
Sham	3.39 ± 0.66	0.22 ± 0.05	3.18 ± 0.62	0.07 ± 0.01

Note: * statistically significant difference from the sham-exposed group (*p* < 0.05 by a Student’s *t*-test).

**Table 2:** Some biochemical indices in the BALF supernatant obtained 24 h after the last 4 h inhalation exposure to NiO-NP at 2.4 ± 0.4 µg/m^3^; (*x* ± *s.e.*).

Index	Exposure Period							
	2 Weeks		4 Weeks		3 Months		6 Months	
	Sham-Exposed	Exposed to NiO-NP	Sham-Exposed	Exposed to NiO-NP	Sham-Exposed	Exposed to NiO-NP	Sham-Exposed	Exposed to NiO-NP
Alkaline phosphatase, U/L	43.32 ± 7.18	54.89 ± 3.24	57.05 ± 4.04	45.07 ± 8.58	18.81 ± 6.54	13.19 ± 2.92	17.11 ± 3.79	17.67 ± 2.13
Aspartate-aminotransferase, U/L	6.48 ± 1.16	7.25 ± 1.29	5.03 ± 0.68	4.40 ± 1.22	4.29 ± 0.88	5.84 ± 1.46	5.77 ± 0.74	4.96 ± 0.52
Alanine-aminotransferase, U/L	0.67 ± 0.18	1.75 ± 0.95	2.09 ± 0.54	2.31 ± 0.36	0.66 ± 0.29	0.31 ± 0.14	0.27 ± 0.14	0.27 ± 0.26
Amilase, U/L	6.32 ± 1.34	6.25 ± 1.81	3.34 ± 0.24	2.87 ± 0.46	1.89 ± 0.27	1.90 ± 0.35	2.47 ± 0.44	2.10 ± 0.21
γ-glutamyl-transpeptidase, U/L	1.87 ± 0.40	2.24 ± 0.55	1.33 ± 0.31	1.23 ± 0.25	1.47 ± 0.53	1.23 ± 0.39	1.91 ± 0.50	2.23 ± 0.22
Glucose, mmol/L	0.01 ± 0.01	0.03 ± 0.02	0.00 ± 0.00	0.01 ± 0.01	0.00 ± 0.00	0.00 ± 0.00	0.00 ± 0.00	0.00 ± 0.00
Lactate dehydrogenase, U/L	35.11 ± 6.10	67.75 ± 17.59	35.50 ± 4.27	31.57 ± 6.22	25.86 ± 8.37	19.57 ± 8.05	27.86 ± 5.78	33.86 ± 5.47

**Table 3:** Some indices of rat organism’s status after repeated inhalation of nickel-oxide nanoparticles (*x* ± *s.e.*).

Duration of Exposure							
2 Weeks		4 Weeks		3 Months		6 Months	
Groups of Rats							
Sham-Exposed (Control))	Exposed to NiO-NP	Sham-Exposed (Control)	Exposed to NiO-NP	Sham-Exposed (Control)	Exposed to NiO-NP	Sham-Exposed (Control)	Exposed to NiO-NP
Indices							
Brain mass, g							
1.91 ± 0.04	1.94 ± 0.03	1.96 ± 0.06	1.86 ± 0.06	2.03 ± 0.03	1.95 ± 0.03	2.07 ± 0.05	1.89 ± 0.06 *
Kidney mass, g							
1.43 ± 0.03	1.37 ± 0.07	1.56 ± 0.03	1.47 ± 0.05	1.91 ± 0.09	1.76 ± 0.06	2.15 ± 0.06	1.95 ± 0.06 *
Reticulocytes, ‰							
5.10 ± 0.64	7.10 ± 0.46 *	7.67 ± 1.41	11.67 ± 1.67	No data	No data	6.17 ± 0.31	16.14 ± 1.40 *
Endogeneous creatinine clearance, mL/24 h							
0.72 ± 0.06	0.68 ± 0.06	0.49 ± 0.07	0.42 ± 0.04	0.58 ± 0.11	0.72 ± 0.06	0.87 ± 0.06	0.55 ± 0.11 *
Blood cell genomic DNA fragmentation coefficient							
0.4235 ± 0.0032	0.4477 ± 0.0041 *	0.4223 ± 0.0019	0.4489 ± 0.0003 *	0.4225 ± 0.0013	0.4535 ± 0.029 *	0.4223 ± 0.0015	0.4623 ± 0.013 *
Leukocyte specific lysis reaction, %							
6.12 ± 1.27	8.32 ± 1.88	4.10 ± 1.06	12.96 ± 1.37 *	No data	No data	No data	No data
Ni-specific IgE							
1.05 ± 0.09	1.22 ± 0.13	0.94 ± 0.16	1.18 ± 0.14	No data	No data	No data	No data
Eosinophils in spleen tissue imprints, %							
4.57 ± 0.65	8.29 ± 1.57 *	5.00 ± 0.37	7.50 ± 1.18	3.22 ± 0.84	5.10 ± 1.28	3.19 ± 0.81	5.33 ± 1.48

* Index is statistically significantly different from the control (*p* < 0.05 by Student’s *t*-test).

## Data Availability

Data available on request due to restrictions eg privacy or ethical

## Supplementary Materials

The following are available online at https://www.mdpi.com/1422-0067/22/1/416/s1, Table S1: Indices for organism’s status measured in control (sham-exposed) and exposed to NiO-NP rats (x ± s.e.), Table S2: Some cytological characteristics of different organ imprints f; percentage of total cell count (x ± s.e.).
