# Supplementary material for: Looking for the LOAEL or NOAEL Concentration of Nickel-Oxide Nanoparticles in a Long-Term Inhalation Exposure of Rats
Source: Int J Mol Sci. 2021 Jan 3;22(1):416. doi: 10.3390/ijms22010416 (PMC7796390; doi:10.3390/ijms22010416)
Supplement: Supplementary file 1 [file ijms-22-00416-s001.pdf]

Supplement Table S1. Indices for organism's status measured in control (sham-exposed) and exposed to NiO-NP rats (x ± s.e.)

Indices	Duration of Exposure							
	2 weeks		4 weeks		3 Months		6 Months	
	Control	NiO-NP	Control	NiO-NP	Control	NiO-NP	Control	NiO-NP
Body mass before exposure, g	230.00 ± 4.48	231.25 ± 5.05	228.14 ± 3.86	222.43 ± 4.68	272.00 ± 5.04	261.29 ± 6.92	272.54 ± 9.13	260.50 ± 8.62
Body mass after exposure, g	242.00 ± 3.49	233.25 ± 4.87	246.29 ± 3.15	241.21 ± 4.35	273.36 ± 6.56	263.57 ± 7.36	288.46 ± 10.23	280.71 ± 9.29
Body mass gain, %	5.49 ± 1.04	**0.95 ± 0.79 ***	8.06 ± 0.61	8.59 ± 1.01	0.50 ± 1.58	0.96 ± 1.60	6.36 ± 3.22	8.11 ± 2.22
Number of head-dips into holes during 3 min	5.20 ± 0.51	5.50 ± 0.52	8.07 ± 1.21	8.79 ± 1.01	7.00 ± 1.10	6.71 ± 0.87	5.38 ± 0.93	6.07 ± 0.79
Number of crossed squares per 3 min	10.85 ± 1.13	10.35 ± 0.94	15.71 ± 1.69	18.14 ± 1.80	12.93 ± 1.57	14.29 ± 1.68	11.38 ± 1.40	12.79 ± 1.22
Total number of movements on the "open field" during 3 min	19.05 ± 1.78	19.15 ± 1.73	27.93 ± 3.08	32.79 ± 3.59	24.86 ± 2.65	26.36 ± 3.12	20.77 ± 2.38	22.36 ± 1.96
Temporal summation of sub-threshold impulses, sec	17.80 ± 0.44	16.07 ± 0.48 *	13.18 ± 0.53	11.82 ± 0.53	8.32 ± 1.13	8.46 ± 0.69	9.45 ± 0.99	11.56 ± 0.84
Brain mass, g	1.91 ± 0.04	1.94 ± 0.03	1.96 ± 0.06	1.86 ± 0.06	2.03 ± 0.03	1.95 ± 0.03	2.07 ± 0.05	1.89 ± 0.06 *
Heart mass, g	0.75 ± 0.02	0.79 ± 0.03	0.83 ± 0.02	0.77 ± 0.02	0.92 ± 0.03	0.90 ± 0.03	1.09 ± 0.03	1.02 ± 0.06
Liver mass, g	7.10 ± 0.19	7.13 ± 0.26	9.09 ± 0.12	8.93 ± 0.31	9.17 ± 0.74	9.28 ± 0.42	10.22 ± 0.69	9.70 ± 0.65
Spleen mass, g	0.45 ± 0.02	0.47 ± 0.02	0.56 ± 0.03	0.50 ± 0.02	0.57 ± 0.03	0.50 ± 0.02	0.66 ± 0.03	0.57 ± 0.03
Kidney mass, g	1.43 ± 0.03	1.37 ± 0.07	1.56 ± 0.03	1.47 ± 0.05	1.91 ± 0.09	1.76 ± 0.06	2.15 ± 0.06	**1.95 ± 0.06 ***
Lung mass, g	1.28 ± 0.06	1.33 ± 0.06	2.07 ± 0.28	1.88 ± 0.19	2.32 ± 0.31	2.41 ± 0.31	2.50 ± 0.33	2.77 ± 0.66
Brain mass , g per 100 g body mass	0.81 ± 0.02	0.86 ± 0.03	0.81 ± 0.03	0.76 ± 0.03	0.75 ± 0.03	0.75 ± 0.03	0.71 ± 0.04	0.68 ± 0.03
Heart mass, g per 100 g body mass	0.33 ± 0.01	0.33 ± 0.02	0.34 ± 0.01	0.32 ± 0.01	0.34 ± 0.01	0.34 ± 0.01	0.37 ± 0.02	0.36 ± 0.02

Indices	Duration of Exposure							
	2 weeks		4 weeks		3 Months		6 Months	
	Control	NiO-NP	Control	NiO-NP	Control	NiO-NP	Control	NiO-NP
Liver mass, g per 100 g body mass	3.14 ± 0.14	3.01 ± 0.10	3.74 ± 0.08	3.66 ± 0.15	3.33 ± 0.21	3.56 ± 0.18	3.41 ± 0.16	3.42 ± 0.18
Spleen mass, g per 100 g body mass	0.20 ± 0.01	0.20 ± 0.01	0.23 ± 0.01	0.20 ± 0.01	0.21 ± 0.01	**0.19 ± 0.01 ***	0.22 ± 0.01	0.20 ± 0.01
Kidney mass, g per 100 g body mass	0.60 ± 0.01	0.60 ± 0.02	0.64 ± 0.01	0.60 ± 0.02	0.70 ± 0.03	0.67 ± 0.03	0.73 ± 0.02	0.69 ± 0.02
Lung mass, g per 100 g body mass	0.54 ± 0.02	0.59 ± 0.03	0.85 ± 0.11	0.76 ± 0.06	0.85 ± 0.10	0.93 ± 0.13	0.86 ± 0.13	1.02 ± 0.28
Hemoglobin, g/dL	148.80 ± 3.57	156.60 ± 6.55	154.00 ± 2.78	148.67 ± 2.76	150.86 ± 2.92	156.57 ± 6.00	176.33 ± 12.37	169.14 ± 7.42
Hematocrit, %	22.74 ± 0.54	24.22 ± 1.01	19.80 ± 0.72	19.28 ± 0.61	20.81 ± 0.63	21.49 ± 0.74	24.52 ± 1.96	24.06 ± 1.12
Reticulocytes, ‰	5.10 ± 0.64	7.10 ± 0.46 *	7.67 ± 1.41	11.67 ± 1.67	-	-	6.17 ± 0.31	16.14 ± 1.40 *
Erythrocytes, 10^{12} cells/L	7.41 ± 0.17	7.82 ± 0.33	7.54 ± 0.18	7.44 ± 0.13	7.05 ± 0.19	7.32 ± 0.22	8.75 ± 0.80	8.44 ± 0.41
Mean corpuscular volume, μm^3.	61.42 ± 0.67	61.97 ± 0.72	52.47 ± 0.78	51.80 ± 0.89	59.04 ± 0.96	58.71 ± 1.25	57.17 ± 0.90	57.13 ± 0.82
Mean corpuscular hemoglobin, 10^{-12} g	20.10 ± 0.19	20.03 ± 0.23	20.47 ± 0.25	19.97 ± 0.24	21.43 ± 0.23	21.03 ± 0.31	20.63 ± 0.29	20.10 ± 0.40
Mean corpuscular hemoglobin concentration, g/L	327.20 ± 2.43	323.20 ± 1.25	390.50 ± 9.40	386.50 ± 8.62	363.14 ± 5.18	358.86 ± 5.79	344.83 ± 19.20	351.86 ± 4.76
Red cell distribution width, %	13.24 ± 0.20	12.97 ± 0.24	11.47 ± 0.20	11.17 ± 0.33	13.33 ± 0.29	12.97 ± 0.17	12.38 ± 0.33	11.97 ± 0.24
Thrombocytes, $10^3/\mu L$	821.60 ± 60.92	823.00 ± 51.90	718.00 ± 34.25	635.33 ± 68.61	706.57 ± 17.88	811.43 ± 45.83	729.00 ± 80.13	814.57 ± 41.19
Mean platelet volume, μm^3	5.50 ± 0.08	5.54 ± 0.07	5.37 ± 0.07	5.55 ± 0.11	6.00 ± 0.14	5.61 ± 0.08	5.83 ± 0.09	5.66 ± 0.08
Thrombocrit, %	0.22 ± 0.02	0.23 ± 0.01	0.19 ± 0.01	0.18 ± 0.02	0.21 ± 0.01	0.23 ± 0.01	0.21 ± 0.02	0.23 ± 0.01
Thrombocyte distribution width, %	12.71 ± 0.51	12.90 ± 0.24	12.45 ± 0.36	13.23 ± 0.21	13.59 ± 0.49	13.80 ± 0.57	14.33 ± 0.32	14.60 ± 0.78
Leukocytes, $10^3/\mu L$	5.64 ± 0.72	6.66 ± 0.63	6.30 ± 0.43	6.43 ± 0.17	7.03 ± 0.59	6.71 ± 1.20	7.93 ± 0.63	7.59 ± 0.74

Indices	Duration of Exposure							
	2 weeks		4 weeks		3 Months		6 Months	
	Control	NiO-NP	Control	NiO-NP	Control	NiO-NP	Control	NiO-NP
Basophils, $10^3/\mu L$	0.00 ± 0.00	0.00 ± 0.00	0.00 ± 0.00	0.00 ± 0.00	0.00 ± 0.00	0.00 ± 0.00	0.00 ± 0.00	0.00 ± 0.00
Eosinophils, $10^3/\mu L$	0.21 ± 0.03	0.44 ± 0.11	0.20 ± 0.04	0.26 ± 0.06	0.40 ± 0.06	0.23 ± 0.06	0.43 ± 0.08	0.47 ± 0.08
Banded neutrophils, $10^3/\mu L$	0.06 ± 0.01	0.08 ± 0.01	0.07 ± 0.01	0.06 ± 0.00	0.07 ± 0.01	0.07 ± 0.01	0.10 ± 0.02	0.08 ± 0.01
Segmented neutrophils, $10^3/\mu L$	1.10 ± 0.13	1.26 ± 0.13	1.36 ± 0.13	1.29 ± 0.07	1.80 ± 0.22	1.59 ± 0.32	0.10 ± 0.02	0.08 ± 0.01
Monocytes, $10^3/\mu L$	0.30 ± 0.03	0.35 ± 0.03	0.32 ± 0.03	0.34 ± 0.01	0.39 ± 0.06	0.45 ± 0.09	1.78 ± 0.17	1.76 ± 0.20
Lymphocytes, $10^3/\mu L$	3.97 ± 0.55	4.52 ± 0.42	4.35 ± 0.31	4.47 ± 0.23	4.37 ± 0.32	4.38 ± 0.74	5.18 ± 0.50	4.84 ± 0.46
Basophils, %	0.00 ± 0.00	0.00 ± 0.00	0.00 ± 0.00	0.00 ± 0.00	0.00 ± 0.00	0.00 ± 0.00	0.00 ± 0.00	0.00 ± 0.00
Eosinophils, %	4.00 ± 0.52	6.00 ± 1.01	3.33 ± 0.61	4.17 ± 1.05	5.71 ± 0.92	3.14 ± 0.51	5.67 ± 1.20	5.57 ± 0.69
Banded neutrophils, %	1.00 ± 0.00	1.20 ± 0.20	1.17 ± 0.17	1.00 ± 0.00	1.00 ± 0.00	1.14 ± 0.14	1.33 ± 0.33	1.00 ± 0.00
Segmented neutrophils, %	20.20 ± 1.23	19.10 ± 1.22	21.50 ± 0.85	20.17 ± 1.25	25.29 ± 1.32	23.00 ± 1.54	22.50 ± 1.36	23.00 ± 0.53
Monocytes, %	5.50 ± 0.31	5.40 ± 0.27	5.00 ± 0.37	5.33 ± 0.21	5.43 ± 0.48	6.57 ± 0.57	5.50 ± 0.22	6.00 ± 0.38
Lymphocytes, %	69.30 ± 1.54	68.30 ± 1.65	69.00 ± 1.21	69.33 ± 2.29	62.57 ± 1.46	66.14 ± 2.10	65.00 ± 2.13	64.00 ± 0.90
Succinate dehydrogenase (SDH) activity, number of formazan granules per 50 lymphocytes	587.00 ± 14.30	618.70 ± 18.01	560.67 ± 5.48	558.00 ± 8.53	582.29 ± 7.39	571.14 ± 6.05	526.67 ± 7.79	519.14 ± 5.54
Total protein content of blood serum, g/L	77.63 ± 1.71	77.05 ± 1.30	77.57 ± 0.73	77.25 ± 0.67	71.70 ± 1.53	70.29 ± 1.28	79.13 ± 1.96	80.46 ± 1.04
Albumin content of blood serum, g/L	45.97 ± 0.53	45.62 ± 0.34	47.72 ± 0.80	46.72 ± 0.92	41.30 ± 0.53	40.19 ± 2.10	43.72 ± 2.20	44.07 ± 1.58
Globulins of blood serum, g/L	31.67 ± 1.44	31.43 ± 1.07	29.85 ± 0.74	30.53 ± 1.15	30.40 ± 1.57	30.10 ± 1.47	35.42 ± 1.88	36.39 ± 1.06

Indices	Duration of Exposure							
	2 weeks		4 weeks		3 Months		6 Months	
	Control	NiO-NP	Control	NiO-NP	Control	NiO-NP	Control	NiO-NP
A/G index	1.47 ± 0.07	1.46 ± 0.04	1.61 ± 0.06	1.54 ± 0.08	1.38 ± 0.07	1.37 ± 0.12	1.26 ± 0.12	1.22 ± 0.07
ALT activity in blood serum, mM/h·L	68.55 ± 3.67	60.20 ± 4.21	60.88 ± 5.25	76.28 ± 12.56	47.13 ± 2.63	57.61 ± 4.55	76.28 ± 8.03	90.20 ± 5.87
AST activity in blood serum, mM/h·L	225.35 ± 14.31	198.53 ± 9.01	355.63 ± 83.13	444.75 ± 64.77	299.73 ± 14.02	340.77 ± 22.04	265.80 ± 29.09	311.79 ± 29.65
De Ritis coefficient	3.31 ± 0.23	3.33 ± 0.13	5.61 ± 0.92	6.00 ± 0.69	6.43 ± 0.37	6.01 ± 0.32	3.50 ± 0.25	3.50 ± 0.34
SH-groups in blood serum, µmol/L	0.20 ± 0.13	0.35 ± 0.13	0.00 ± 0.00	0.12 ± 0.00	-	-	-	-
Activity of γ-glutamintransferase in blood serum, nmol/(s·L)	1.68 ± 0.79	0.65 ± 0.14	5.98 ± 1.87	7.75 ± 2.73	0.53 ± 0.34	0.57 ± 0.30	2.62 ± 0.71	2.73 ± 0.69
Alkaline phosphatase in blood serum, nmol/(s·L)	109.38 ± 9.67	120.47 ± 16.71	140.13 ± 6.30	139.62 ± 21.21	119.96 ± 18.69	85.21 ± 11.42	124.15 ± 21.32	113.19 ± 15.08
Lactate dehydrogenase (LDH), U/L	2564.17 ± 244.88	2481.17 ± 180.42	2968.33 ± 328.87	3306.67 ± 167.41	3691.43 ± 237.78	4010.00 ± 162.91	3333.33 ± 250.01	3498.57 ± 135.86
Amilase in blood serum, U/L	2730.83 ± 135.12	2685.67 ± 80.98	4633.67 ± 384.02	4678.33 ± 411.86	3285.57 ± 274.66	3187.00 ± 348.41	3339.33 ± 369.49	3378.00 ± 227.76
Catalase in blood serum, µmol/L	0.35 ± 0.03	0.38 ± 0.02	0.44 ± 0.04	0.40 ± 0.03	1.90 ± 0.22	2.16 ± 0.11	0.62 ± 0.05	0.60 ± 0.07
Reduced glutathione in the blood hemolysate, µmol/L	30.37 ± 4.78	31.86 ± 8.76	21.54 ± 1.34	22.34 ± 1.68	19.16 ± 1.27	20.04 ± 1.22	21.05 ± 1.66	21.51 ± 1.69
Ceruloplasmin in blood serum, mg/%	54.40 ± 5.26	57.86 ± 5.24	34.25 ± 7.27	31.58 ± 6.21	62.70 ± 12.14	86.37 ± 11.39	68.38 ± 5.18	82.73 ± 7.91
MDA in blood serum, nmol/L	4.36 ± 0.61	4.50 ± 0.60	2.29 ± 0.34	3.19 ± 0.45	3.94 ± 0.15	4.74 ± 0.38	3.85 ± 0.23	4.31 ± 0.43
Uric acid in blood serum, µmol/L	78.00 ± 15.04	92.33 ± 4.18	117.67 ± 15.51	126.83 ± 8.92	133.00 ± 10.37	111.14 ± 12.74	-	-

Indices	Duration of Exposure							
	2 weeks		4 weeks		3 Months		6 Months	
	Control	NiO-NP	Control	NiO-NP	Control	NiO-NP	Control	NiO-NP
Uric acid in urine, μmol/L	55.80 ± 4.15	66.30 ± 16.34	56.83 ± 6.91	64.33 ± 3.11	100.27 ± 12.37	83.92 ± 18.43	-	-
Urea in blood serum, mmol/L	5.38 ± 0.26	5.65 ± 0.49	4.20 ± 0.36	4.88 ± 0.37	2.89 ± 0.66	2.86 ± 0.25	6.43 ± 0.48	6.00 ± 0.41
Urea in urine, mmol/L	121.57 ± 8.10	101.63 ± 10.49	105.84 ± 5.73	117.04 ± 5.32	133.64 ± 8.09	125.97 ± 19.02	81.61 ± 5.36	77.11 ± 4.20
Creatinine in blood serum, μmol/L	39.02 ± 1.89	35.78 ± 1.93	39.02 ± 2.58	38.60 ± 1.44	45.34 ± 2.70	45.50 ± 2.05	45.33 ± 2.15	51.36 ± 2.88
Creatinine in urine, mmol/L	0.69 ± 0.05	0.82 ± 0.15	0.64 ± 0.04	0.72 ± 0.05	1.01 ± 0.09	1.03 ± 0.14	0.97 ± 0.05	**0.73 ± 0.03 ***
Endogenous creatinine clearance, ml/min	0.72 ± 0.06	0.68 ± 0.06	0.49 ± 0.07	0.42 ± 0.04	0.58 ± 0.11	0.72 ± 0.06	0.87 ± 0.06	**0.55 ± 0.11 ***
Bilirubin in blood serum, μmol/L	1.02 ± 0.08	1.07 ± 0.13	1.07 ± 0.11	1.32 ± 0.12	1.76 ± 0.18	1.34 ± 0.11	1.97 ± 0.21	1.69 ± 0.12
Cholesterol in blood serum, mmol/L	2.48 ± 0.10	2.31 ± 0.13	2.11 ± 0.16	2.35 ± 0.09	2.15 ± 0.17	2.10 ± 0.12	2.08 ± 0.22	2.48 ± 0.25
High density lipoproteins in blood serum, mmol/L	2.13 ± 0.09	2.00 ± 0.11	1.69 ± 0.09	1.86 ± 0.08	1.87 ± 0.15	1.84 ± 0.13	1.74 ± 0.16	2.07 ± 0.18
Low density lipoproteins in blood serum, mmol/L	0.23 ± 0.02	0.24 ± 0.03	0.21 ± 0.03	0.22 ± 0.02	0.48 ± 0.06	0.42 ± 0.04	0.33 ± 0.05	0.47 ± 0.06
Triglycerides in blood serum, mmol/L	1.10 ± 0.15	0.92 ± 0.05	1.31 ± 0.08	1.38 ± 0.15	0.68 ± 0.03	**0.85 ± 0.07 ***	0.68 ± 0.10	0.65 ± 0.04
Diuresis, mL	35.80 ± 1.68	31.10 ± 2.05	29.67 ± 3.59	22.29 ± 2.37	25.50 ± 3.80	33.42 ± 2.26	35.25 ± 3.88	40.42 ± 2.34
Urine pH	8.05 ± 0.16	7.65 ± 0.13	7.42 ± 0.08	7.33 ± 0.11	6.91 ± 0.06	7.04 ± 0.07	7.27 ± 0.10	7.04 ± 0.04
Urine relative density, g/mL	1.02 ± 0.00	1.02 ± 0.00	1.01 ± 0.00	1.02 ± 0.00	1.01 ± 0.00	1.02 ± 0.00	1.01 ± 0.00	1.01 ± 0.00
Protein in urine, g/L	47.35 ± 6.79	51.92 ± 14.45	52.47 ± 9.15	52.77 ± 5.83	75.37 ± 7.26	95.03 ± 9.72	-	-

Indices	Duration of Exposure							
	2 weeks		4 weeks		3 Months		6 Months	
	Control	NiO-NP	Control	NiO-NP	Control	NiO-NP	Control	NiO-NP
Total coproporphyrin in urine, µmol	56.93 ± 9.02	59.56 ± 7.53	13.91 ± 5.20	31.23 ± 8.27	37.76 ± 10.81	41.38 ± 7.54	74.83 ± 20.27	105.26 ± 33.18
Daily coproporphyrin in urine, µmol	1.70 ± 0.27	2.25 ± 0.42	0.36 ± 0.10	1.50 ± 0.61	1.46 ± 0.38	1.46 ± 0.40	2.07 ± 0.61	2.92 ± 0.90
δ-aminolevulinic acid (ALA) in urine, µg/mL	15.33 ± 3.93	15.46 ± 4.02	-	-	14.04 ± 3.00	15.02 ± 5.72	3.94 ± 1.04	3.39 ± 0.86
Daily δ-aminolevulinic acid (ALA) in urine, µg	0.49 ± 0.15	0.55 ± 0.15	-	-	0.65 ± 0.17	0.45 ± 0.15	0.10 ± 0.03	0.09 ± 0.02

Note: * statistically significant difference from the control group; $p < 0.05$ by the Student's t-test with Bonferroni correction.

Supplement Table S2. Some cytological characteristics of different organ imprints f; percentage of total cell count (x ± s.e.)

Organs and cells	Duration of Exposure							
	2 weeks		4 weeks		3 Months		6 Months	
	Control (sham-exposure)	NiO-NP	Control (sham-exposure)	NiO-NP	Control (sham-exposure)	NiO-NP	Control (sham-exposure)	NiO-NP
Lungs								
Neutrophils	6.71 ± 0.75	7.71 ± 1.66	6.33 ± 0.49	8.17 ± 1.72	10.71 ± 1.44	8.86 ± 0.70	6.33 ± 0.49	6.71 ± 0.75
Degeneratively changed neutrophils	4.71 ± 0.92	3.14 ± 0.40	1.83 ± 0.31	3.17 ± 0.48 *	4.71 ± 0.68	6.43 ± 0.72	3.50 ± 0.43	4.71 ± 0.92
Alveolar macrophages	5.00 ± 0.93	6.14 ± 0.59	9.83 ± 0.75	7.00 ± 0.97 *	3.43 ± 0.20	2.86 ± 0.40	5.00 ± 0.73	5.00 ± 0.93
Degeneratively changed alveolar macrophages◎	6.00 ± 0.82	17.57 ± 1.25 *	2.50 ± 0.56	19.17 ± 3.03 *	9.14 ± 1.10	16.14 ± 2.24 *	10.33 ± 1.73	6.00 ± 0.82
Bronchial epithelial cells	18.14 ± 1.26	7.00 ± 0.76 *	17.67 ± 0.88	8.67 ± 0.49 *	9.14 ± 1.32	12.86 ± 1.37	13.83 ± 1.58	18.14 ± 1.26

Organs and cells	Duration of Exposure							
	2 weeks		4 weeks		3 Months		6 Months	
	Control (sham-exposure)	NiO-NP	Control (sham-exposure)	NiO-NP	Control (sham-exposure)	NiO-NP	Control (sham-exposure)	NiO-NP
Degeneratively changed bronchial epithelial cells	4.71 ± 0.29	4.29 ± 0.61	3.83 ± 0.48	3.67 ± 0.76	4.00 ± 0.31	9.00 ± 0.87 *	5.67 ± 0.71	4.71 ± 0.29
Lymphocytes	53.43 ± 3.06	53.14 ± 3.52	56.67 ± 1.48	45.50 ± 1.52 *	56.57 ± 2.76	42.00 ± 2.08 *	51.00 ± 3.60	53.43 ± 3.06
Eosinophils	1.29 ± 0.18	1.00 ± 0.22	1.67 ± 0.33	0.83 ± 0.31	2.71 ± 0.57	1.86 ± 0.40	3.33 ± 0.76	1.29 ± 0.18
Lymphatic nodes								
Mature lymphocytes, prolymphocytes	90.33 ± 1.05	87.29 ± 0.99	92.50 ± 0.62	90.80 ± 0.97	88.71 ± 0.99	84.29 ± 0.57 *	89.17 ± 1.01	87.00 ± 1.38
Lymphoblasts	1.50 ± 0.34	1.86 ± 0.40	1.33 ± 0.21	1.20 ± 0.20	1.29 ± 0.18	1.57 ± 0.20	1.33 ± 0.21	1.40 ± 0.24
Reticular cells	0.67 ± 0.21	0.29 ± 0.18	0.00 ± 0.00	0.00 ± 0.00	0.43 ± 0.20	0.71 ± 0.18	0.33 ± 0.21	0.20 ± 0.20
Plasmocytes	2.67 ± 0.42	5.29 ± 0.61 *	1.67 ± 0.49	2.60 ± 0.51	5.29 ± 0.92	5.57 ± 0.48	3.83 ± 0.60	5.20 ± 1.16
Macrophages	1.50 ± 0.34	1.57 ± 0.30	1.50 ± 0.22	2.40 ± 0.24 *	1.71 ± 0.29	3.43 ± 0.30 *	2.00 ± 0.26	2.20 ± 0.20
Neutrophils	2.33 ± 0.76	3.14 ± 0.46	1.83 ± 0.31	2.00 ± 0.77	1.57 ± 0.37	2.71 ± 0.29 *	2.17 ± 0.48	2.40 ± 0.40
Eosinophils	1.00 ± 0.00	0.71 ± 0.18	1.17 ± 0.17	1.00 ± 0.00	1.00 ± 0.22	1.71 ± 0.29	1.17 ± 0.17	1.60 ± 0.40
Spleen								
Mature lymphocytes, prolymphocytes	85.00 ± 0.65	78.29 ± 1.61 *	83.00 ± 0.89	81.67 ± 2.06	70.53 ± 7.58	65.25 ± 7.52	69.37 ± 8.48	63.48 ± 8.59
Lymphoblasts	1.14 ± 0.14	1.71 ± 0.29	1.33 ± 0.42	1.00 ± 0.26	1.01 ± 0.14	1.02 ± 0.16	1.33 ± 0.18	1.16 ± 0.18
Reticular cells	0.29 ± 0.18	0.29 ± 0.18	0.33 ± 0.21	0.33 ± 0.21	0.52 ± 0.14	0.58 ± 0.11	0.52 ± 0.16	0.29 ± 0.12
Plasmocytes	1.29 ± 0.29	1.57 ± 0.37	1.33 ± 0.21	1.33 ± 0.21	3.16 ± 0.58	3.31 ± 0.54	2.51 ± 0.40	3.20 ± 0.62
Macrophages	2.29 ± 0.18	3.14 ± 0.40	1.83 ± 0.31	3.33 ± 0.67	1.72 ± 0.17	2.48 ± 0.31	1.63 ± 0.18	1.70 ± 0.22

Organs and cells	Duration of Exposure							
	2 weeks		4 weeks		3 Months		6 Months	
	Control (sham-exposure)	NiO-NP	Control (sham-exposure)	NiO-NP	Control (sham-exposure)	NiO-NP	Control (sham-exposure)	NiO-NP
Neutrophils	5.43 ± 0.30	7.00 ± 0.90	7.17 ± 0.79	4.67 ± 1.09	4.67 ± 1.19	6.46 ± 1.85	4.32 ± 0.95	5.87 ± 1.47
Eosinophils	4.57 ± 0.65	8.29 ± 1.57 *	5.00 ± 0.37	7.50 ± 1.18	3.22 ± 0.84	5.10 ± 1.28	3.19 ± 0.81	5.33 ± 1.48
Liver								
Duct epithelial cells	7.00 ± 0.53	9.29 ± 0.57 *	6.83 ± 0.75	7.50 ± 0.76	9.71 ± 1.17	7.71 ± 0.57	9.50 ± 0.72	7.33 ± 0.67
Hepatocytes	69.29 ± 0.64	52.14 ± 3.38 *	66.67 ± 1.28	52.00 ± 4.48 *	59.86 ± 1.64	57.14 ± 3.04	67.67 ± 1.26	58.17 ± 2.47 *
Degeneratively changed hepatocytes◎	6.86 ± 0.51	15.71 ± 2.68 *	9.33 ± 1.31	15.33 ± 1.63 *	7.00 ± 1.07	14.00 ± 0.62 *	5.83 ± 0.54	13.17 ± 0.40 *
Neutrophils	4.43 ± 0.48	9.29 ± 1.15 *	5.83 ± 1.14	12.33 ± 1.73 *	12.29 ± 0.99	10.14 ± 1.49	9.00 ± 0.73	9.67 ± 1.17
Eosinophils	6.43 ± 0.69	8.00 ± 0.90	5.33 ± 1.48	7.67 ± 1.52	5.14 ± 0.59	6.43 ± 1.19	3.83 ± 0.31	6.83 ± 1.33
Binucleated hepatocyte	1.43 ± 0.20	1.00 ± 0.00	1.50 ± 0.22	1.17 ± 0.17	1.29 ± 0.18	0.86 ± 0.14	1.33 ± 0.21	1.00 ± 0.00
Kupffer cells	3.29 ± 0.36	4.00 ± 0.53	3.33 ± 0.42	3.33 ± 0.49	3.71 ± 0.61	3.14 ± 0.34	1.83 ± 0.31	3.17 ± 0.17 *
Fibroblasts	1.29 ± 0.18	0.57 ± 0.20 *	1.17 ± 0.31	0.67 ± 0.21	1.14 ± 0.26	0.57 ± 0.20	1.00 ± 0.00	0.67 ± 0.21
Kidney								
Proximal tubule cells	61.57 ± 1.19	51.29 ± 1.69 *	59.17 ± 1.89	48.67 ± 3.44 *	60.00 ± 1.89	59.57 ± 0.72	60.50 ± 1.45	54.33 ± 1.09 *
Degenerated cells of proximal tubules	7.14 ± 0.77	15.14 ± 0.70 *	7.00 ± 0.97	16.67 ± 1.86 *	6.14 ± 1.03	10.14 ± 0.40 *	4.67 ± 0.42	14.83 ± 0.70 *
Distal tubule cells	11.86 ± 1.03	8.57 ± 1.11	12.67 ± 1.12	9.00 ± 1.21	12.86 ± 0.83	7.57 ± 0.53 *	13.17 ± 0.79	7.50 ± 0.62 *
Degenerated cells of distal tubules	5.14 ± 0.40	11.29 ± 0.64 *	4.83 ± 0.48	9.33 ± 0.80 *	4.86 ± 0.40	6.71 ± 0.42 *	4.83 ± 0.48	6.83 ± 0.60 *

Organs and cells	Duration of Exposure							
	2 weeks		4 weeks		3 Months		6 Months	
	Control (sham-exposure)	NiO-NP	Control (sham-exposure)	NiO-NP	Control (sham-exposure)	NiO-NP	Control (sham-exposure)	NiO-NP
Neutrophils	7.86 ± 0.59	6.71 ± 0.52	7.67 ± 0.61	6.67 ± 0.61	7.57 ± 0.53	6.29 ± 0.64	7.33 ± 0.76	7.33 ± 0.80
Monocytes	4.71 ± 0.57	5.57 ± 0.65	4.67 ± 0.49	6.00 ± 0.58	4.86 ± 0.40	5.14 ± 0.51	6.17 ± 0.48	4.67 ± 0.21 *
Eosinophils	1.29 ± 0.18	1.29 ± 0.18	3.67 ± 0.76	3.50 ± 0.22	3.43 ± 0.61	4.29 ± 0.36	3.00 ± 0.52	4.33 ± 0.49
Fibroblasts	0.43 ± 0.20	0.14 ± 0.14	0.33 ± 0.21	0.17 ± 0.17	0.29 ± 0.18	0.14 ± 0.14	0.33 ± 0.21	0.17 ± 0.17

Note: * statistically significant difference from the control group; $p < 0.05$ by the Student's t-test with Bonferroni correctioin.
